# Determination of Serotonin and Dopamine Metabolites in Human Brain Microdialysis and Cerebrospinal Fluid Samples by UPLC-MS/MS: Discovery of Intact Glucuronide and Sulfate Conjugates

**DOI:** 10.1371/journal.pone.0068007

**Published:** 2013-06-27

**Authors:** Tina Suominen, Päivi Uutela, Raimo A. Ketola, Jonas Bergquist, Lars Hillered, Moshe Finel, Hongbo Zhang, Aki Laakso, Risto Kostiainen

**Affiliations:** 1 Division of Pharmaceutical Chemistry, Faculty of Pharmacy, University of Helsinki, Helsinki, Finland; 2 Centre for Drug Research, Faculty of Pharmacy, University of Helsinki, Helsinki, Finland; 3 Analytical Chemistry and Neurochemistry, Department of Chemistry – BMC and Science for Life Laboratory, University of Uppsala, Uppsala, Sweden; 4 Neurosurgery, Department of Neuroscience, University of Uppsala, Uppsala, Sweden; 5 Department of Neurosurgery, Helsinki University Central Hospital, Helsinki, Finland; Biological Research Centre of the Hungarian Academy of Sciences, Hungary

## Abstract

An UPLC-MS/MS method was developed for the determination of serotonin (5-HT), dopamine (DA), their phase I metabolites 5-HIAA, DOPAC and HVA, and their sulfate and glucuronide conjugates in human brain microdialysis samples obtained from two patients with acute brain injuries, ventricular cerebrospinal fluid (CSF) samples obtained from four patients with obstructive hydrocephalus, and a lumbar CSF sample pooled mainly from patients undergoing spinal anesthesia in preparation for orthopedic surgery. The method was validated by determining the limits of detection and quantification, linearity, repeatability and specificity. The direct method enabled the analysis of the intact phase II metabolites of 5-HT and DA, without hydrolysis of the conjugates. The method also enabled the analysis of the regioisomers of the conjugates, and several intact glucuronide and sulfate conjugates were identified and quantified for the first time in the human brain microdialysis and CSF samples. We were able to show the presence of 5-HIAA sulfate, and that dopamine-3-O-sulfate predominates over dopamine-4-O-sulfate in the human brain. The quantitative results suggest that sulfonation is a more important phase II metabolism pathway than glucuronidation in the human brain.

## Introduction

**Figure 1:**
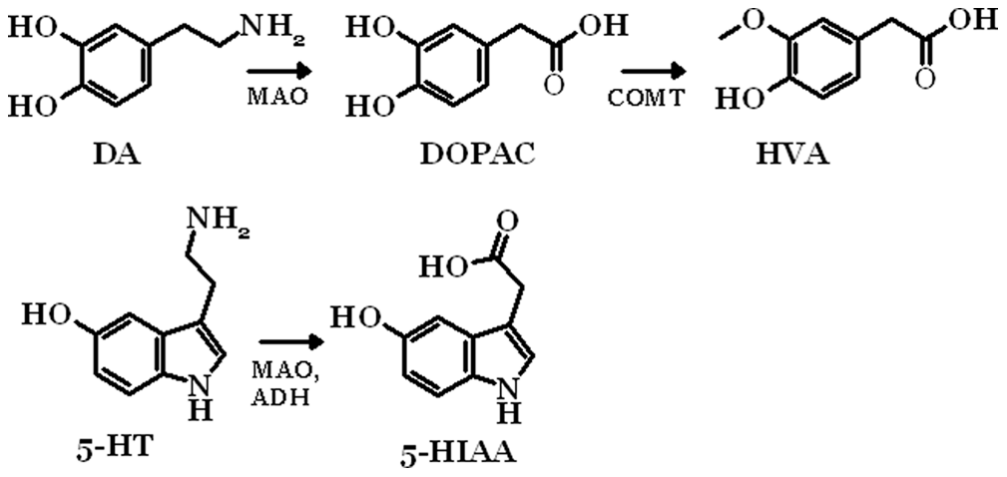
Main metabolic routes of serotonin and dopamine to their phase I metabolites.

Dopamine (DA) and serotonin (5-HT) are monoamine neurotransmitters in the human brain that are involved in several physiological processes. 5-HT is involved in the regulation of several physiological functions, including the sleep-wake cycles, body temperature, blood pressure, perception of pain, hormonal functions of the hypothalamus and psychological functions, such as depression and anxiety [1], [2]. The functions of DA have been linked to Parkinson’s disease, schizophrenia, depression and the regulation of motoric movements [3]–[5]. The levels of both these neurotransmitters are regulated in the brain by reuptake and metabolism. Both DA and 5-HT are metabolized by monoamine oxidase (MAO) to the phase I metabolites 3,4-dihydroxyphenylacetic acid (DOPAC) and 5-hydroxyindoleacetic acid (5-HIAA), respectively. DOPAC is further metabolized to homovanillic acid (HVA) by catechol-O-methyltrasferase (COMT) (Figure 1). Both DA and 5-HT, and their respective metabolites, can undergo conjugation with glucuronic acid or sulfonate mediated by catalysis with UDP-glucuronosyltransferases (UGTs) and sulfotransferases (SULTs), respectively. The UGTs are a family of enzymes that catalyze the glucuronidation of various compounds, and thereby have an important role in the intestinal, hepatic and renal metabolism and detoxification of a large number of xenobiotic and endogenous compounds [6], [7]. The expression of UGTs is tissue-specific. Many UGTs are expressed in the liver, but UGTs are also expressed in other organs and some of the UGTs are expressed only or mainly in extra-hepatic tissues, such as the gastrointestinal tract, the olfactory mucosa, adipose tissue and the kidneys. Small amounts of UGT mRNA have also been found in several other tissues, including the heart, adrenal gland, trachea and brain [7]–[10]. In the human brain, which is the focus of this study, low mRNA levels of the UGTs 1A4, 1A5, 1A6, 2A1, 2A2, 2A3, 2B7, 2B11 and 2B17 have been reported. However, the findings were not consistent, which was probably due to the low number of donors and inter-individual variability [8], [10]. In addition, some of the earlier studies on UGTs expression in different organs [6], [7] were carried out using less quantitative methods that are more prone to errors. Recent studies used quantitative real-time PCR (qRT-PCR), in which the detected mRNA levels of the UGTs in the brain samples were either not found or if so were barely above the detection limits. Nevertheless, 5-HT has been shown to be a substrate for at least one human UGT, UGT1A6, and very low 5-HT glucuronidation activity was also reported for UGT2B7 [6], [11], [12]. DA has been shown to be conjugated mainly by UGT1A10, and very low activities were reported for UGTs 1A6, 2A1, 2A3, 2B7, 2B11 and 2B17 [13].

Another family of enzymes, the SULTs, catalyze the sulfonation of different compounds, and are equally important in the metabolic conjugation of xenobiotics and endogenous compounds. There are 13 different human sulfotransferases that can be divided into three families, SULT1, SULT2, and SULT4. Similar to that of the UGTs, the expression of SULTs is tissue-specific. The liver is the most abundant site for most SULTs expression, although the intestine, lung, kidney and brain also express SULTs [14], [15]. SULT 1A3, which is the SULT enzyme that is mainly responsible for monoamine metabolism, in addition to SULT 1A1, 2A1, 1E1 and 4A1, have also been shown to be expressed in the human brain [14], [16].

The phase I metabolism of monoamine neurotransmitters in the human central nervous system is relatively well documented. 5-HT, DA and their phase I metabolites have previously been determined in human lumbar and ventricular cerebrospinal fluid (CSF), and in human brain tissue in several studies [17]–[23]. However, the phase II metabolism in the central nervous system has been less studied, which is partly due to the lack of commercially available standard compounds, i.e. neurotransmitter glucuronides and sulfates. Therefore, such conjugates in the human cerebrospinal fluid (CSF) or brain samples have mostly been analyzed after acid or enzymatic hydrolysis of the conjugates [24]–[28]. Sulfate conjugates of DA, 5-HT, epinephrine and norepinephrine [27]–[30] and glucuronide conjugates of DA and norepinephrine [27] have been identified after hydrolysis of the conjugates in CSF samples. Some studies have reported the detection of conjugated DA, DOPAC, HVA and 5-HT in human CSF, but do not specify the type of conjugate [26], [31]–[33]. Moreover, conjugated monoamine neurotransmitter metabolites have been found in human brain tissue samples. Glucuronide conjugates of HVA, DOPAC, 5-HIAA, and 4-hydroxy-3-methoxy-phenylglycol (a metabolite of norepinephrine) and DOPAC sulfate were identified after hydrolysis in a tissue sample taken from the human caudate nucleus [24]. Furthermore, conjugated DA has been found in the human caudate nucleus and hypothalamus, although the specific conjugate type was not identified [34]. DA and 5-HT were shown to be sulfonated also in *vitro studies* using phenol sulfotransferase preparate isolated from human brain tissue homogenates [35]–[37].

The conjugates of monoamine neurotransmitter in human CSF or brain samples have been analyzed in all the earlier studies by using indirect methods employing either acid or enzymatic hydrolysis of the conjugates. Indirect methods, however, do not provide identification of the conjugates with absolute certainty, since the hydrolysis step is prone to errors even by employing specific enzymes such as β-glucuronidase or sulfatase. In addition, the site of conjugation cannot be determined by indirect methods, and the reliability of the quantification of the conjugates is questionable, since the hydrolysis might not be complete. Only recently, methods have been developed for the analysis of intact phase II metabolites of monoamine neurotransmitters and neurosteroids in rat and mouse brain [38]–[41]. Intact glucuronide conjugates of both 5-HT and DA, and sulfate conjugates of their phase I metabolites 5-HIAA, DOPAC and HVA were recently found in rat and mouse brain microdialysates in our laboratory by using a direct analysis method [40], [41].

Microdialysis, which has been largely used in the analysis of neurotransmitters and their metabolites in the central nervous system of laboratory animals, mainly in rats and mice, provides a more reliable method to examine the physiological state of the brain than the methods employing post mortem brain tissue samples. Microdialysis of the human brain has been used to monitor neurointensive care patients with subarachnoid hemorrhage, traumatic brain injury, thromboembolic stroke or epilepsy [42]–[45]. Monoamine neurotransmitters and their phase I metabolites in human brain microdialysates have been analyzed in only a few studies [43], [44], [46]–[50].

Until now, no intact conjugated phase II metabolites of neurotransmitters have been analyzed in human brain samples and therefore the aim of this work was to study the existence of monoamine neurotransmitter metabolites, especially glucuronides and sulphates, in human brain microdialysates and CSF. For this purpose, we developed a sensitive and selective ultra-performance liquid chromatographic - mass spectrometric (UPLC-MS/MS) method, which provides direct and thus more reliable analysis of sulfate and glucuronide conjugates than the earlier methods employing hydrolysis of the conjugates.

## Results and Discussion

### UPLC-MS/MS Method Development and Validation

The UPLC-MS/MS method developed in this work for the analysis of DA and 5-HT and their phase I and phase II metabolites (Table S1) in human brain microdialysis and CSF samples was based on the HPLC-MS/MS method presented earlier by our group [40], [41]. Pentafluorophenyl columns were used in both studies, but the column is narrower (2.1 mm) and the particle size smaller (1.9 µm) in the UPLC method than in the HPLC method (4 mm, 3 µm). The advantage of using a narrow UPLC column instead of a normal size HPLC column was the possibility to reduce the injection volume from 100 µL to 15 µL, without reducing sensitivity. Relatively high injection volumes were necessary in order to achieve sufficient sensitivity levels.

The use of a more hydrophilic pentafluorophenylpropyl column instead of a commonly used C-18 column provided good retention and separation of the metabolites from each other and also from the inorganic salts in the Ringer’s solution. The salts were directed to waste using column switching before the elution of the analytes, in order to avoid contamination of the ion source, thus ensuring long-term robust analysis. The UPLC method provided high separation resolution for the analytes, and also separation of the regioisomers DA-3-O-sulfate (DA-3-O-S) and DA-4-O-sulfate (DA-4-O-S), in addition to HVA-O-glucuronide (HVA-O-G) and HVA-COO-glucuronide (HVA-COO-G) (Figures S1 and S2). Electrospray ionization (ESI) in the positive ion mode provided high ionization efficiency for 5-HT, DA, 5-HT-glucuronide (5-HT-G), 5-HT-sulfate (5-HT-S), and DA-glucuronide (DA-G), whereas the negative ion mode provided better ionization efficiency for DA-3-O-S and DA-4-O-S, as well as for DOPAC, HVA, 5-HIAA, and their glucuronide and sulfate conjugates. The positive and negative ion ESI mass spectra showed abundant [M+H]^+^ and [M–H]^−^ ions, which were chosen for the precursor ions. The MS and MS/MS spectra have been presented and discussed earlier [41]. The identification of the neurotransmitters and their metabolites was based on the comparison of the retention times and relative abundances of 2–3 selected reaction monitoring (SRM) transitions (Table S1) of each analyte between the reference standards diluted in Ringer’s solution and the authentic samples. The variation of the intensity ratios of the monitored product ions was less than 15%, and the variation of the retention times was less than 1.5% in all positive identifications. The samples were analyzed in two runs using the same chromatographic gradient but monitoring different SRM transitions in order to maximize sensitivity and selectivity.

The quantitative UPLC-MS/MS method was validated for the following parameters: specificity, limit of detection (LOD) with a signal-to-noise ratio (S/N) of 3, limit of quantification (LOQ) with a S/N of at least 10 and at most a 20% deviation from the linearity curve, linearity, and repeatability using standards diluted in Ringer’s solution (Table 1). LODs were determined by analyzing low concentration spiked standard samples and blank samples (n = 5). The measured low concentration samples resulted in signals of which S/N values varied between 3 and 10. The LODs were estimated by calculating the concentration of the respective analytes that would give a S/N-ratio of 3. The specificity of the method was tested and verified by comparing blank and spiked Ringer’s solution samples. The SRM chromatograms of the blank samples (Ringer’s solution) showed no analyte peaks, which indicated good specificity and no memory effects. The LODs for 5-HT, DA, 5-HT-G, 5-HT-S and DA-G analyzed in the positive ion mode were 0.02–0.40 nM (0.3–6 fmol injected on the column). The LODs for DA-3-O- and DA-4-O-S, HVA, DOPAC, 5-HIAA, and their glucuronides and sulfates in the negative ion mode varied between 0.3 and 25 nM (4.5–375 fmol injected on the column). The LODs for DA and 5-HT and their metabolites were at the same level as described earlier [39]–[41], [51], [52]. The correlation coefficients (r^2^) for the calibration curves that were measured by using an external standard method were >0.996, which indicated good linearity of the method. The within-day repeatability of the method was studied at concentration levels of 10 and/or 100 nM (Table 1). The relative standard deviations (% rsd) were below 10%, indicating good repeatability. Concerning the microdialysis samples, the recoveries of the neurotransmitters and their phase I and II metabolites across the microdialysis membrane were not determined, but the typical in vivo recovery for small molecules (glucose, lactate, pyruvate, glutamate) in microdialysis has been reported to be 65–70% [53]. The concentration ratios between the analytes are expected to be similar to those in the brain fluid, since the permeability of the analytes through the microdialysis membrane is not selective.

**Table 1:** Validation results for all the compounds analyzed**.**

	Linearity		LOD	LOQ		Repeatability	Repeatability
	range		S/N = 3	S/N ≥10	Accuracy	10 nM, n = 6	100 nM, n = 6
Compound	nM	r2	nM	nM	%	rsd %	rsd %
**DA**	0.5–25	0.997	0.2	0.5	90–120	6.0	nm
**DA-G**	0.5–50	0.998	0.1	0.5	90–110	4.3	nm
**DA-3-S**	1–50	0.997	0.3	1	91–116	5.1	0.6
	50-1000	0.998			93–115		
**DA-4-S**	1–1000	0.998	0.3	1	91–112	5.7	1.5
**5-HT**	1–25	0.996	0.2	1	89–112	1.8	nm
**5-HT-G**	0.25–25	0.997	0.02	0.25	96–103	2.5	nm
**5-HT-S**	1–100	0.999	0.4	1	94–107	2.6	nm
**DOPAC**	50–1000	0.998	10	50	95–110	nm	
**DOPAC-G**	25–1000	0.996	2.5	25	93–113	nm	2.0
**DOPAC-S**	25–1000	0.998	4.3	25	95–110	nm	nm
**HVA**	100–1000	0.999	25	100	98–102	nm	7.0
	250–5000	0.999			95–120		
**HVA-OH-G**	5–100	0.999	1.0	5	92–111	4.9	2.9
**HVA-S**	5–500	0.999	1.4	5	95–118	nm	nm
**5-HIAA**	25–1000	0.998	6.4	25	91–106	nm	4.0
	250–3500	0.999			95–103		
**5-HIAA-G**	5–500	0.998	1.0	5	86–116	7.2	3.2
**5-HIAA-S**	10–1000	0.999	5.0	10	96–113	nm	10.0

*nm* = not measured.

### Neurotransmitters and their Metabolites Measured in the Human Brain Microdialysis and CSF Samples

**Figure 2:**
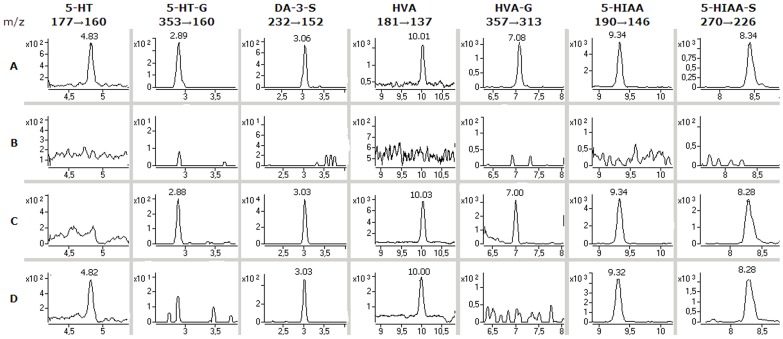
Findings in the human brain microdialysis samples. SRM ion chromatograms of A: standards (1 nM 5-HT and 5-HT-G, 5 nM DA-3-O-S and HVA-O-G, 100 nM HVA, 5-HIAA and 5-HIAA-S); B: a blank sample (Ringer’s solution); C: a human brain microdialysis sample from patient 1; D: a human brain microdialysis sample from patient 2.

The microdialysis samples from two patients (patients 1 and 2) suffering from acute brain injury (subarachnoid hemorrhage) were analyzed as such. 5-HT was identified at low concentrations (<1 nM) in the microdialysis samples from patient 2, but not from patient 1. DA was not detected in any of the samples (Table 2). The LODs for 5-HT and DA were 0.2 nM respectively (Table 1), which suggests that the concentrations of 5-HT (patient 1) and DA (both patients) were below their respective LODs. The most abundant metabolite of DA was HVA (150–2000 nM), but HVA-O-glucuronide (12–15 nM) was also identified in the samples obtained from patient 1. DA-3-O-S was clearly identified in both patients (patient 1∶330–630 nM, patient 2∶2.5–3.5 nM), but DA-4-O-S was not found in any of the samples. The most abundant metabolites of 5-HT were 5-HIAA (90–110 nM) and 5-HIAA-S (380–550 nM), but low concentrations of 5-HT-G (0.45–0.8 nM) were also identified in the samples from patient 1 (Figure 2).

**Table 2:** Findings in the human brain microdialysis and the human cerebrospinal fluid samples.

Microdialysis samples					Cerebrospinal fluid samples				
	Patient 1		Patient 2		Ventricular				Lumbar
Compound	Sample 1	Sample 2	Sample 1	Sample 2	Patient 3	Patient 4	Patient 5	Patient 6	Pooled
**5-HT**	–	–	0.52^a^	0.54^a^	0.84^a^	–	0.70^a^	1.2	–
**5-HT-S**	–	–	–	–	–	–	–	–	0.63^a^
**5-HT-G**	0.45	0.80	–	–	–	–	–	–	–
**5-HIAA**	90	110	90	100	1400	340	220	540	35
**5-HIAA-S**	450	550	520	380	370	170	96	390	86
**5-HIAA-G**	–	–	–	–	–	–	–	–	–
**DA**	–	–	–	–	–	–	–	–	–
**DA-3-O-S**	330	630	3.5	2.5	10	18	14	9.4	12
**DA-4-O-S**	–	–	–	–	–	–	2.0	–	–
**DA-G**	–	–	–	–	–	–	–	–	–
**HVA**	1100	2000	280	150	4200	601	2200	2200	120
**HVA-S**	–	–	–	–	–	–	–	–	–
**HVA-O-G**	15	12	–	–	–	–	–	–	–
**DOPAC**	–	–	–	–	–	–	–	–	–
**DOPAC-S**	–	–	–	–	–	–	–	–	–
**DOPAC-G**	–	–	–	–	–	–	–	–	–

All concentrations are in nM.

The microdialysis samples (patients n = 2) were analyzed as singles. Each of the CSF samples (four ventricular CSF samples (patients 1–4), one lumbar CSF sample pooled from several patients were analyzed in triplicate (concentrations shown are the mean concentrations). – not detected, ^a^<LOQ, concentration estimated by extrapolation of the linearity curve.

**Figure 3:**
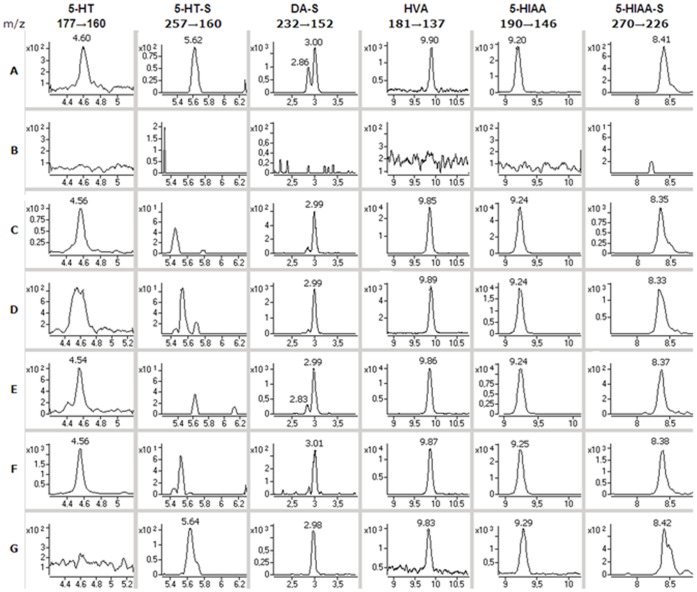
Findings in the human CSF samples. SRM ion chromatograms of A: standards (0.5 nM 5-HT and 5-HT-S, 10 nM DA-4- and 3-O-S and 100 nM HVA, 5-HIAA and 5-HIAA-S); B: a blank sample (Ringer’s solution); C-F: ventricular cerebrospinal fluid samples from patients 3–6; G: lumbar cerebrospinal fluid sample pooled from several patients.

Low concentrations of 5-HT (1.2 nM or less) were detected in three of the ventricular CSF samples similar to those found in the brain microdialysis samples. In comparison DA was not detected in any of the samples. The most abundant metabolite of DA was also HVA, but the concentrations of which were about one order of magnitude higher (600–4200 nM) in the ventricular samples than in the lumbar sample (120 nM). DA-3-O-S (9.4–18 nM) was detected in all the samples but DA-4-O-S only in one sample (2.0 nM). The most abundant metabolites of 5-HT were 5-HIAA (35–1400 nM) and 5-HIAA-S (86–390 nM), which corresponded to those found for the microdialysis samples. 5-HT-S was detected at a low concentration only in the lumbar CSF sample, but 5-HT-G that was detected in the microdialysis samples was not detected in any of the CSF samples (Figure 3).

The findings and concentrations of the neurotransmitters and their metabolites are in relatively good agreement between the microdialysis and the CSF samples. The main phase I metabolites of DA and 5-HT were HVA and 5-HIAA, respectively. These results are consistent with earlier studies of their concentrations in human brain tissue [18]–[22] and human CSF samples [17], [18], [29], [54]. Earlier studies reported that DOPAC was detected in human brain tissue samples [19], [21], [22], [34], in microdialysis samples [46], [48] and CSF samples [17], [18], [29], but it was not detected in this study. This may be due to insufficient sensitivity of the method or to fast degradation of DA and DOPAC during the sample storage, as reported earlier by several groups [28], [40], [55].

The intact phase II metabolites, 5-HIAA-S, DA-3-O-S, DA-4-O-S, 5-HT-S, 5-HT-G, and HVA-O-G were detected in human brain or CSF for the first time in this work. There are earlier reports of HVA-G, DOPAC-G, 5-HIAA-G and DOPAC-S in tissue samples of the human brain [24], and DA-G, 5-HT-G, 5-HT-S [27] and DA-S [27]–[30], [33], [54] have been found in human CSF. However, all these metabolites were detected after a hydrolysis step, which makes their determinations less reliable than our direct method of detecting intact conjugates. In addition 5-HIAA-S, which has not been detected in the human brain or CSF in earlier works, was now found at relatively high concentrations. The concentrations of 5-HIAA-S were around 3–5 times higher than those of free 5-HIAA in the human microdialysis and lumbar CSF samples, and about half the concentration of free 5-HIAA in the ventricular CSF samples.

DA-3-O-S was clearly detected in all the samples and DA-4-O-S in only one sample, which indicated that the DA-3-O-S regioisomer predominates in the human brain. These results are in accordance with the observation that DA-3-O-S predominates in human plasma at concentrations of about ten-fold higher than those of the regioisomer DA-4-O-S [56]. It has been shown that the isoenzyme SULT1A3, which is also found in the human brain [14], is regioselective and strongly favors the formation of DA-3-O-S over DA-4-O-S [57]. Sulfonation of 5-HT was less favored than sulfonation of DA, as 5-HT-S was only detected in the lumbar CSF sample at a low concentration.

Two glucuronides, 5-HT-G and HVA-O-G, were detected for the first time in human brain samples. However, the glucuronides were only found in the microdialysis samples of patient 1 at low concentrations, and no glucuronides were detected in the CSF samples. The amount of HVA-O-G was less than 1% of the amount of free HVA. This result may be considered to be in line with the reported 5-HT glucuronidation of UGT1A6 (Figure S3 and ref. 11) and the barely detectable expression of this enzyme in the brain [6], [8]. However, this result is not in line with another study that reported on 23 tissue types [10]. HVA can be glucuronidated both at the phenolic hydroxyl and the carboxylic acid group sites. Our screening experiment reveals that UGT1A10 is the most active human UGT in the formation of HVA-O-glucuronide, whereas UGT2A1 is the most active for the conjugation of HVA at the –COOH-group (Fig. S4). These glucuronidation results are interesting and new, but they also clearly show that glucuronidation is not a major biotransformation pathway for 5-HT and HVA in the human brain. In general, the results in Table 2 suggest that conjugation with sulfonate is the major phase II metabolism pathway in the human brain.

### Conclusions

The microdialyis sampling and the developed UPLC-MS/MS-analysis method require minimal sample pretreatment, and provide sensitive and selective analysis of intact phase II metabolites of neurotransmitters in the human brain. In this study, intact phase II metabolites of 5-HT and DA were analyzed in the human brain samples without hydrolysis of the conjugates, and several intact glucuronides and sulfates were unambiguously identified and quantified in human brain microdialysis and CSF samples. 5-HIAA-S was found for the first time in human brain. As the direct method provides analysis of regioisomers of conjugates, we were able to show that DA-3-O-S predominates over DA-4-O-S in the human brain. The quantitative results show that sulfonation is a significant phase II metabolism pathway in the human brain.

Further studies including samples from a larger group of patients would be needed to study interindividual differences in the amounts of conjugated neurotransmitters versus the concentrations of the parent compounds. The metabolic turnover of the compounds should also be studied. The role of the conjugation of neurotransmitter in the brain is still unknown. It is also unknown whether the conjugates possess neurotoxic or neuroprotective properties, are pharmacologically active or not, or even if they are hydrolysed *in vivo*, and additional experiments are needed in order to study the pharmacological impact of conjugated and especially sulfonated neurotransmitters on different brain functions.

## Materials and Methods

### Reagents and Standards

3,4-dihydroxyphenethylamine hydrochloride (DA) and 3-methoxy-4-hydroxyphenylacetic acid (HVA) were purchased from Sigma-Aldrich (St. Louis, MO). 5-hydroxytryptamine (5-HT), 3,4-dihydroxyphenylacetic acid (DOPAC) and 5-Hydroxyindole-3-acetic acid (5-HIAA) and formic acid were purchased from Sigma-Aldrich (Steinheim, Germany). Acetonitrile (ACN) was purchased from Rathburn (Walkerburn, Scotland). Ringer’s solution, used for dilution of the standards, contained 147 mM NaCl (Akzo Nobel, Denmark), 1.2 mM CaCl_2_ (J.T.Bakers, Deventer, The Netherlands), 2.7 mM KCl (Sigma-Aldrich, Steinheim, Germany), 1.0 mM MgCl_2_ (Riedel-de-Haehn AG, Seelze, Germany), and 0.04 mM ascorbic acid (University Pharmacy, Helsinki, Finland). Uridine 5′-diphosphoglucuronic acid tri-ammonium salt (UDPGA), were purchased from Sigma–Aldrich (St. Louis, MO, USA).

### Synthesis of Phase II Metabolites

The synthesis of the phase II metabolites of 5-HT, DA and their phase I metabolites, which were used as reference standards in this study, had been done earlier in our laboratory and has been described in detail elsewhere [40], [41].

### Microdialysis Samples

The human brain microdialysis samples were obtained from the Neurointensive care unit of the Uppsala University Hospital, in collaboration with the University of Uppsala, from two patients with acute brain injuries (subarachnoid hemorrhage). 172 fractions were collected from patient 1. The sample volume was about 5–10 µL per fraction, collected in a time-resolved mode from day 1–8. 132 fractions of similar volume were collected from patient 2 in a time-resolved mode from day 1–6. Both patients were female, aged 71 and 50 years, had a decreased level of consciousness, were intubated and received artificial ventilation. Propofol sedation was used initially. Intracerebral microdialysis sampling was initiated, in conjunction with the insertion of an ICP monitoring device, through microdialysis catheters inserted via a bur hole placed 1–2 cm anterior to the coronal suture. Care was taken to insert the microdialysis probe obliquely in macroscopically nonlesioned cortex by using atraumatic techniques aiming at an intracortical positioning of the microdialysis membrane. Microdialysis catheters with a membrane length of 10 mm and a 20-kDa nominal molecular weight cut-off polyamide membrane (70 Brain Microdialysis Catheter; M Dialysis AB, Solna, Sweden) were used. The outflow hydrostatic pressure of the perfusion system was set at the zero mid-cranial reference level by taping the collecting vials next to the bandage on the patient’s head. Perfusion of the catheters was performed using artificial CSF (Perfusion Fluid CNS, M Dialysis AB), containing NaCl 147 mM, CaCl_2_ 1.7 mM, KCl 2.7 mM, MgCl 0.85 mM, total chloride contents 153.8 mM, osmolarity 305 mOsm/kg), delivered at a rate of 0.3 µL/min by using a microdialysis pump (106 MD Pump, M Dialysis AB). At least 2 hours passed between insertion of the probe and the start of sampling to allow for normalization of changes due to probe insertion. The samples were frozen on dry ice and stored at -70°C, and were thawed and pooled just before the analysis. The samples were injected as such and analyzed as single replicates. The sampling was approved by the Regional Research Ethics Committee at Uppsala University, and a written informed consent was obtained from the patient or the patient's closest relative, in case the patient was unconscious.

### Cerebrospinal Fluid Samples

The human ventricular cerebrospinal fluid samples (n = 4) were obtained from the Department of Neurosurgery at Helsinki University Central Hospital. The samples were obtained by ventriculostomy, and approximately 10 mL of ventricular CSF was obtained from each patient. The patients were being treated in the neurosurgical intensive care unit for obstructive hydrocephalus. The patients were a 82-year old male with a cerebellar infarct (patient 3), a 46-year old female with subarachnoid hemorrhage (patient 4), a 35-year old male with a cerebellar infarct (patient 5), and a 55-year old female with a tumor (patient 6). Since the samples were taken from CSF waste accumulated during therapeutic CSF drainage (appr. 200 ml/day/patient), which was going to be discarded as a part of the clinical routine, no informed consent from the patients or the next of kin was deemed necessary.

A pool of CSF from 200 subjects from Sweden (all without a neurologic or psychiatric disease, most who underwent lumbar puncture for non-diagnostic reasons and who had normal CSF clinical laboratory values; over 90% were undergoing spinal anesthesia in preparation for orthopedic surgery (e.g. limbs as knees and hips) [58]. Ages ranged from 16 to 65 years with a median of 44 years; 50∶50 female:male. These samples were collected on ice and cells were removed by centrifugation [59]. Approval for the conduct of this study was obtained from the Institutional Review Board in accordance with federal regulations. Approval for the conduct of this study was obtained from the local Ethics Committee at Uppsala University as well as Göteborg University, Sweden. The participants provided their written informed consent to participate in this study. A written informed consent was obtained from the next of kin, caretaker, or guardian on the behalf of participants that were not able to sign the informed consent themselves. The ethics committees approved of this consent procedure.

All cerebrospinal fluid samples were kept at -70°C until analysis. After thawing, the samples were ultrafiltered (Millipore Amicon Ultrafree-MC, 30 000 NMWL) by centrifugation (12 000 g, 15 min), and the filtrate was injected as such. All the CSF samples were analyzed in triplicate.

### UGT Experiments

All UGTs had been expressed in our laboratory as described earlier [12], [60], [61]. Glucuronidation activities were determined with 2 mM 5-HT or HVA, and 5 mM UDPGA, 50 mM phosphate buffer pH 7.4 and 10 mM MgCl_2_ and the samples were incubated at 37°C for 60 min. All UGTs were screened as duplicates, except for 5-HT as a substrate for 2B15, which was analyzed as a single sample and showed no activity. Negative control samples, including all the reaction assay components, with the exception of UDPGA, were also analyzed.

### UPLC-MS/MS

The UPLC-MS/MS system used in the analysis consisted of an Aquity UPLC (Waters, Milford, MA) coupled to an Agilent 6410 triple quadrupole (Agilent technologies, Santa Clara, CA). The column used was a pentafluorophenyl column (Thermo Scientific Gold PFP Hypersil, 2,1 x 150 mm, 1.9 µm). The eluents used were A: aqueous 0.1% formic acid and B: acetonitrile +0.1% formic acid. Different linear gradients were used for the microdialysis and cerebrospinal fluid samples. The gradient used in the analysis of the microdialysis samples was: B 3% 0–1.5 min, 15% 12 min, 65% 13 -13.2 min, 3% 13.5 min, followed by an equilibration of 11.5 min with a total run time of 25 minutes. The flow rate was 0.3 mL/min, and the column oven temperature was kept at 30°C. The injection volume was 15 µl. The gradient used for the analysis of the cerebrospinal fluid samples was similar: B 3% 0–1.5 min, 15% 12 min, 85% 13 -13.2 min, 3% 13.5 min, followed by an equilibration of 11.5 min with a total run time of 25 minutes. The flow from the UPLC was directed to waste for the first minutes of the gradient (Table S1) to avoid contamination of the ion source by the salts of the Ringer’s solution or the cerebrospinal fluid.

An Agilent 6410 triple-quadrupole mass spectrometer (Agilent Technologies, Santa Clara, CA) with an electrospray ion source was used in the quantitative analyses of the brain samples. Nitrogen (Parker Balston N2-22 nitrogen generator, Parker Hannifin Corporation, Haverhill) was used as the nebulizer (40 psi), curtain (12 L/min, 350°C), and collision gas. The fragmentor voltages and collision energies were optimized for each compound, and Agilent MassHunter software versions B.04.00 (quantitative data analysis) and B.03.01 (qualitative data analysis) were used for data acquisition and processing. The compound-specific mass spectrometric parameters had been optimized earlier [41].

As the analysis method contained several analytes, which require either positive or negative ionization, two separate runs were needed for each sample. The compounds followed in each run, in addition to the SRM transitions and ionization modes are shown in Table S1. The method was validated in terms of linearity, limit of detection (LOD), limit of quantification (LOQ) and repeatability for each compound.

## Supporting Information

Figure S1 **SRM ion chromatograms of the monoamine neurotransmitters and their metabolites.** (Run 1, see Table S1).
(TIF)

Figure S2 **SRM ion chromatograms of the monoamine neurotransmitters and their metabolites.** (Run 2, see Table S1).
(TIF)

Figure S3 **5-HT glucuronidation by different UGT enzymes**. The actual and normalized glucuronidation rates (with respect to the expression level of UGT1A10) of the enzymes are shown. These data represent the means of duplicate samples. See material and methods for details.
(EMF)

Figure S4 **HVA glucuronidation at the phenolic and the carboxylic acid groups by different UGT enzymes**. The actual and normalized glucuronidation rates with respect to the expression level of UGT1A10 of the enzymes are shown. See material and methods for details.
(EMF)

Table S1 **Time segments of the chromatographic runs, SRM transitions and ionisation modes of the analytes.**
(XLSX)
