# Supplementary figures and images for: Determination of Serotonin and Dopamine Metabolites in Human Brain Microdialysis and Cerebrospinal Fluid Samples by UPLC-MS/MS: Discovery of Intact Glucuronide and Sulfate Conjugates
Source: PLoS One. 2013 Jun 27;8(6):e68007. doi: 10.1371/journal.pone.0068007 (PMC3694921; doi:10.1371/journal.pone.0068007)

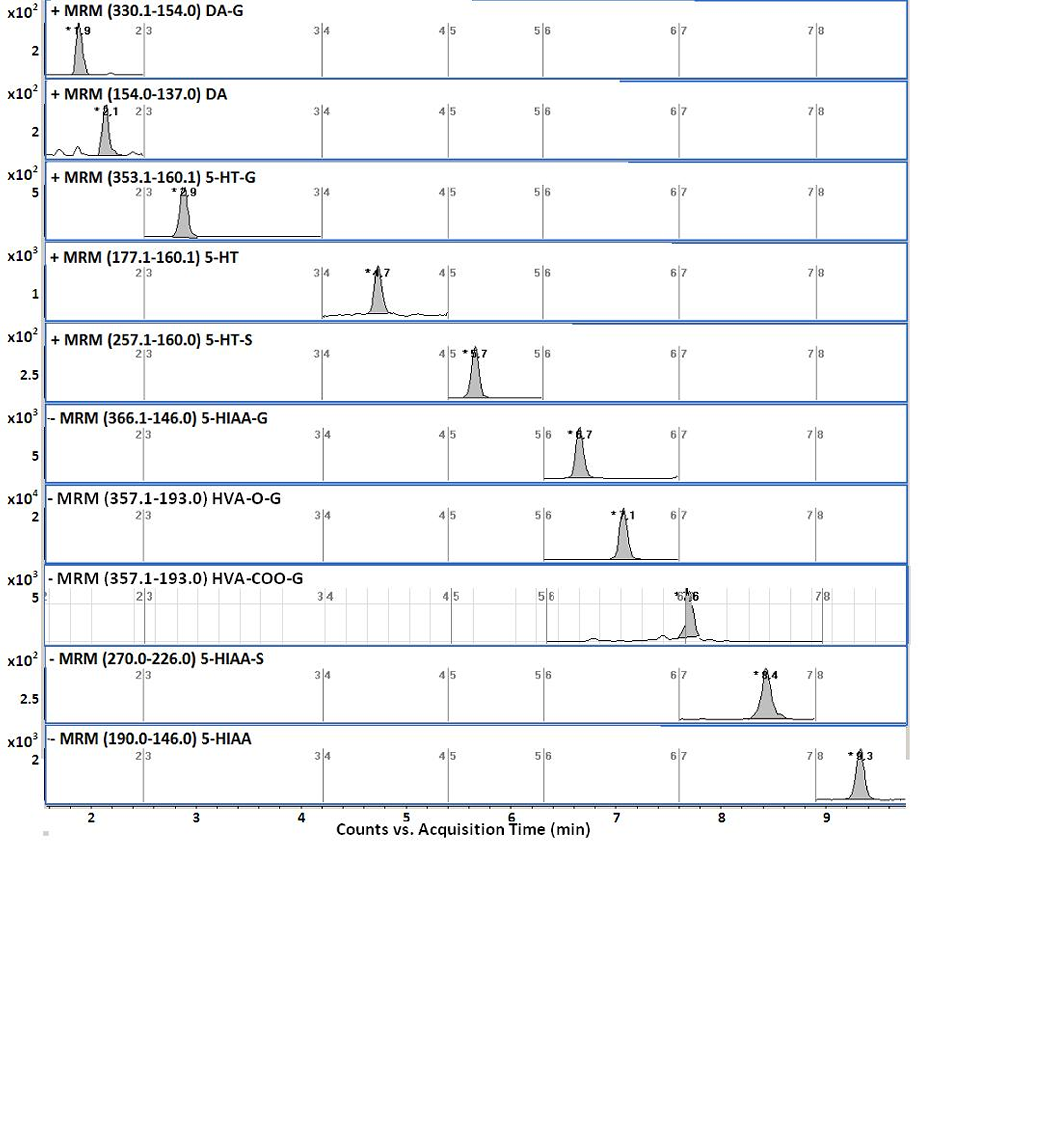

Supplement: Figure S1 — SRM ion chromatograms of the monoamine neurotransmitters and their metabolites. (Run 1, see Table S1). (TIF) [file pone.0068007.s001.tif]
